# Improving the genetic potential of okra (*Abelmoschus esculentus* L.) germplasm to tolerate salinity stress

**DOI:** 10.1038/s41598-023-48370-4

**Published:** 2023-12-06

**Authors:** Ikram ul Haq, Noman Azam, Muhammad Ashraf, Muhammad Mansoor Javaid, Ghulam Murtaza, Zeeshan Ahmed, Muhammad Asam Riaz, Rashid Iqbal, Muhammed Habib ur Rahman, Mona S. Alwahibi, Mohamed S. Elshikh, Muhammad Usman Aslam, Muhammad Arslan

**Affiliations:** 1 Department of Plant Breeding and Genetics, College of Agriculture, University of Sargodha, Sargodha, 40100 Punjab Pakistan; 2 Department of Soil and Environmental Sciences, College of Agriculture, University of Sargodha, Sargodha, 40100 Punjab Pakistan; 3 Department of Agronomy, College of Agriculture, University of Sargodha, Sargodha, 40100 Punjab Pakistan; 4 Faculty of Environmental Science and Engineering, Kunming University of Science and Technology, Kunming, 650093 Yunnan China; 5 Department of Agronomy, University of Agriculture Faisalabad, Faisalabad, 38000 Punjab Pakistan; 6 College of Life Science, Shenyang Normal University, Shenyang, 110034 China; 7 Department of Entomology, College of Agriculture, University of Sargodha, Sargodha, 41000 Punjab Pakistan; 8 Department of Agronomy, Faculty of Agriculture and Environment, The Islamia University of Bahawalpur, Bahawalpur, 63100 Pakistan; 9 Institute of Crop Science and Resource Conservation (INRES), Faculty of Agriculture, University of Bonn, Bonn, Germany; 10 Department of Seed Science and Technology, Institute of Plant Breeding and Biotechnology, MNS-University of Agricultural, Multan, Pakistan; 11 Department of Botany and Microbiology, College of Science, King Saud University, 11451 Riyadh, Saudi Arabia

**Keywords:** Plant breeding, Plant development

## Abstract

Okra (*Abelmoschus esculentus* L.) is the most consumed vegetable worldwide with the potential for diverse ecological adaptation. However, increasing salinization and changing climatic conditions are posing serious threats to the growth, yield, and quality of okra. Therefore, to mitigate increasing soil salinization and ensure sustainable okra production under rapidly changing climatic conditions, evaluation of new okra germplasm to develop salt tolerant cultivars is direly needed. The present study was designed to evaluate the genetic resources of okra genotypes for salt tolerance at growth and reproductive phases. Based on mophological and physio-biochemical responses of plants under stress condition, genotypes were divided into salt tolerant and succeptible groups. The experiment was comprised of 100 okra genotypes and each genotype was grown under control conditions and 6.5 dS m^−1^ NaCl concentration in a pot having 10 kg capacity. The experiment was conducted in a completely randomized design and each treatment was replicated three times. The results showed vast genetic variability among the evaluated okra germplasm traits like days to emergence, pod length, pod diameter, plant height, stem girth, and other yield-related parameters. Correlation analysis showed a highly significant positive association among the number of leaves at first flower and plant height at first flower.Likewise, pod weight also revealed a highly significant positive relationship for pod weight plant^-1^, pod length, and K^+^: Na^+^. Principal Component Analysis (PCA) revealed that out of 16 principal components (PCs), five components showed more than one eigenvalue and the first six PCs contributed 67.2% of the variation. Bi-plot analysis illustrated that genotypes 95, 111, 133, 99, and 128, under salt stress conditions, exhibited both high yield per plant and salt-tolerant behavior in other yield-related traits. On the basis of all studied traits, a salt susceptible group and a salt-tolerant group were formed. The salt tolerant group comprised of 97, 68, 95, 114, 64, 99, 111, 133, 128, and 109 genotypes, whereas, the salt susceptible group contained 137, 139, 130, 94, and 125 genotypes. Salt-tolerant okra genotypes were suggested to be used in further breeding programs aimed to develop salt tolerance in okra. These insights will empower precision breeding, underscore the importance of genetic diversity, and bear the potential to address the challenges of salt-affected soils while promoting broader agricultural resilience, economic prosperity, and food security.

## Introduction

Vegetables being a rich source of minerals and vitamins, while low in calories and free of fat are playing a vital role not only in human nutrition and health but also in the livelihood of the farming community in rural and urban areas^1^. Among vegetables, okra (*Abelmoschus esculentus* L.) is an important dietary fiber crop that is also a national vegetable of Pakistan. Due to its low calorie content and richness in potassium (K), magnesium (Mg), vitamins A, C, and antioxidants, okra is known to enhance the immune system and mitigate the risks of heart disease, diabetes, cancer, and stroke^2^. According to Purkait et al.^3^, okra contains lectin protein, that inhibits cancer cell growth in human beings. Moreover, okra helps to bind cholesterol and thus helps to control cholesterol levels in human being.

Okra also plays an important role in world food security. The pods of okra have highly nutritive and much more medicinal value i.e. protein-carbohydrate and vitamin C concerning other vegetables similar to cucurbits, tomatoes, and eggplant^4^. Mukhtar^5^ mentioned that one hectare of vegetables produces high amounts of calories than cereals. Okra is recognized as a perfect villager’s vegetable due to its vigorous behavior, and exclusive percentage of seed protein meaningful fiber content. Okra leaves are used for the feed of cattle, and mucilage is used for the medicinal purpose^6^.

Okra being sensitive to salinity is facing a severe loss in its yield and quality under increasing salinization in the arid and semi-arid environments^7, 8^. Around 20% of the world's cultivated land is affected by varying levels of salt which is a big threat to world food security and safety^9, 10^. All important glycophytic crops under moderate salinity conditions (EC 4.8 dS m^−1^), reduce average yields by 50% − 80%^11^. In this situation feeding global popultion which is expected to reach 9.1 billion by 2050 is a big challenge. It requires to enhance global food production by about 70%^11^. Moreover, estimates suggest that global economic losses due to soil salinization are around US $ 27.3 billion per year^12^. The major causes of increasing salinization and sodification are salty parent material, use of brackish water, wastewater re-utilization, and high evapotranspiration^13–15^. High levels of salts in the rhizosphere of salt-affected soils caused an increase in osmotic pressure due to which water movement from soil to plants is inhibited, declining plant growth because of osmotic stress. According to Munns^16^, increasing salt concentration in the soil causes roots injury, reduces their capacity to absorb water and nutrients, and creates osmotic stress, specific ion toxicity, nutrient imbalance, and oxidative damage, all these are major mechanisms of salt toxicity in plants. Salinity also hampers plant physiology because the varying concentrations of salts in soil solution may interfere with plant metabolism by disturbing enzymatic activities and hormonal balance^1, 17, 18^. Salt stress negatively influences the ion trasnportation and causes the accumulation of toxic sodium ions (Na +) in plant cells, which disrupts ion homeostasis, especially the Na^+^/K^+^ balance^19^. It decreases nitrate, potassium and water contents, while increases the root to shoot ratio. The stress also inhibited the synthesis of nitrogen compounds by reducing nitrate reduction and ammonia assimilation^20^. In addition, high salt concentration resulted in protein breakdown due to the increased proteolytic activity and ammonia levels. When the concentration of sodium exceeds the threshold that the cells can tolerate, it creates a stressful situation with high cytosolic Na^+^^21^. This triggers the release of stress hormones (abscisic acid, ethylene, salicylic acid, and jasmonic acid) in plants, which help them build strong defense systems^22^. At the same time, the levels and signaling of the growth hormones (auxin, gibberellin, cytokinins, and brassinosteroids) that are unfavored are significantly reduced. This results in a slower plant growth while the focus shifts towards building defenses^21^.

For optimum growth and yield, okra requires well-drained loam soil having 6 to 6.75 pH, rich in nutrients and organic matter but low in salt contents^23, 24^. Okra is sensitive to salinity at all growth stages, particularly at the germination stage. Yakoubi et al.^25^reported that salinity stress delayed the germination process, leading to a marked decline in the growth and pod yield of okra. Khajeh-Hosseini et al.^26^ reported that inhibiting effects of salinity on seed germination might be due to osmotic shock or toxicity of ions, particularly sodium (Na +) and/or chloride (Cl−). Abid et al.^23^ have grown okra with saline-sodic water and found that net photosynthesis, transpiration, growth, and pod weight were decreased with increasing salinity. According to Naqve et al.^8^ salinity stress increased the generation of reactive oxygen species (ROS) and caused oxidative stress which damaged the macromolecules, decreasing the okra growth and yield. Abbas et al.^27^ demonstrated that salinity induced osmotic stress and nutrient imbalances in okra, leading to a marked decline in growth, yield and physiological characteristics. According to Habib et al.^28^, due to higher osmotic potential, particularly in the rhizosphere of salt-affected soils, okra seeds have to use more for water extraction and absorption which reduced the seed germination and seedling establishment under salinity stress.

Salt tolerance in okra is the outcome of various processes and factors working at cellular levels within the plant^27, 29^. Different strategies including genetic engineering, biochemical, physiological, morphological and ionic/nutritional adaptations have been employed to induce salt tolerance in okra^7, 30, 31^. Ayub et al. [7] reported that gibberellic acid and potassium silicate stimulated the antioxidant system in okra, and improved the plant’s ability to tolerate salinity. Habib et al.^28^ demonstrated that salt tolerance of okra can significantly be improved by inoculating the seedlings with plant growth-promoting rhizobacteria. Sergeeva et al.^32^ reported that genetic engineering is an important strategy to introduce salt-tolerant plant species and varieties.

"Extensive documentation supports the notion that plant species and varieties within a species can vary significantly in their ability to tolerate salinity^27, 33^. Different plant species and varieties have varying ability to tolerate salinity. This could be attributed to their capacity to accumulate Na^+^/K^+^, exclude salt ions, distribute salt evenly within the plant body, synthesize osmolytes, adjust osmotically, possess a strong antioxidant system or retain more water^23^. In Pakistan, there is a dire need to identify and develop the vegetable species and varieties that are resistant/tolerant to salinity to meet the food demands of the rapidly increasing population. Given the rising salinization rates and increasing vegetable demand in Pakistan, this study aims to investigate the salt tolerance potential of okra (*Abelmoschus esculentus* L.) genotypesand identify genotypes suitable for further breeding programs. This would help to develop salt tolerant okra cultivars which will facilitate sustainable okra cultivation without compromizing the yield and improve farm income.

## Results and discussion

### Growth and yield characteristics

Genetic diversity in plant breeding is a major tool that could also influence the salt tolerance mechanism of okra lines. The salt concentration exhibited a negative correlation with the ontogeny of the breeding accessions, growth and yield characteristics and variation in the concentration of Na^+^, K^+^ and K^+^/Na^+^ ratio. The okra genotypes were also graded for their relative tolerance to NaCl salinity (6.5 dS m^−1^). Salinity stress had highly significant (*P* ≤ 0.01) and variable effects on growth and yield characteristics in terms of DTE, DTF, plant height at first flower (PFF), No. of leaves at first flower (NL), No. of pods per plant (NP), fresh pod weight (PW), fresh pod length (PL) and fresh pod diameter (PD) (Table 1), leaf area (LA) and plant height in cm (PH) (Table 2) of all growing okra genotypes. The difference between genotypes (G) × treatment (T) interaction (G × T) showed a highly significant response at *P* ≤ 0.01 which revealed that all genotypes responded differently under saline conditions (Table 1). The present research agreed with Mahajan and Tuteja^34^ who concluded that saline condition delayed germination and increased DTE which subsequently had a negative effect on the plant growth and yield. Ouis et al.^35^ also found that firstly okra emergence was shown in control and then in saline stress environment. The delayed emergence in okra under salinity stress was associated with salinity-induced osmotic shock which reduced water absorption by seeds, and delayed the breakage of seed coat and thus germination^36^. Water is vital for the breakage of seed coat before germination. The genotypes exhibited significant differences (*P* ≤ 0.01), while treatment (NaCl concentration) showed non-significant effects (*P* ≥ 0.05) on DTF. The genotypic response showed distinct differences under different concentrations of G × T which were also significant at *P* ≤ 0.05. Similar findings were reported by Javaid et al.^37^, Wasaya et al.^38^, Waheed et al.^39^ and Tanveer et al.^40^ under saline conditions. Differential behavior of okra genotypes to DTF was attributed to their abilities to Na^+^, K^+^ and Na^+^: K^+^ ratio. NaCl salinity had a significant (*P* ≤ 0.01) influence on the number of leaf at first flower in okra accession. Genotypes and treatment of NaCl showed significant variable relation with each other. The genotypes also differed significantly (*P* ≤ 0.01) for plant height. The relationship between genotypes and treatment also remained significant for plant height which suggested that okra genotypes behaved differently to NaCl salinity, accessions reacted differently by altering the concentration of NaCl. Naqve et al.^8^ reported that salinity stress reduced cell division and cell elongation, leading to reduced plant height. Since, okra genotypes accumulated different amounts of salt ions hence showed a differential decline in plant height.

**Table 1:** Mean square values for different growth and yield characteristics of okra (*Abelmoschus esculentus* L.) grown under salinity stress.

S.o.V	DTE	DTF	NL	HFF	NP	PL	PD	PW
Genotypes (G)	23.81**	173.47**	4.007**	20.85**	5.27**	2.76**	4.14**	327.0**
Treatment (T)	638.60**	59.53	304.76**	1236.60**	447.20**	217.93**	262.54**	36,109.3**
G × T	9.67**	105.38**	1.32**	1.03**	2.23**	0.96**	1.46**	105.2**
Error	3.75**	18.69**	0.48**	1.88**	0.89**	0.34**	0.28**	34.2**

DTE: Days to emergence; DTF: Days to flowering; NL: No. of leaves at first flower; HFF: Plant height at first flower; NP: No. of pods per plant; PL: Fresh pod length; PD: Fresh pod diameter; PW: Fresh pod weight.**= Significant at 0.01 and 0.05 level.

**Table 2:** Mean square values for different growth, physiological and ionic characteristics of okra (*Abelmoschus esculentus* L.) grown under salinity stress.

S.o.V	PD	RWC	EL	LA	PH	Na^+^	K^+^	K^+^: Na^+^
Genotypes (G)	8.92**	482.5**	166.2**	7,872,997**	292.1**	45.43**	743.1**	38.95**
Treatment (T)	609.15**	42,072.7**	14,738.8**	4.53E + 08**	19,006.3**	1228.17**	16,017.9**	1240.65**
G × T	3.10**	163.3**	50.6**	2,458,496**	82.9**	11.06**	40.7**	6.92**
Error	0.74**	42.8**	10.2**	580,486**	29.8**	0.64**	20.5**	0.87**

PD: Fresh pod diameter; RWC: Relative water content; EL: Electrolyte leakage; LA: Leaf area; PH: Plant height; Na^+^: Sodium; K^+^: Potassium; K^+^: Na^+^: Potassium: sodium ratio. **= Significant at 0.01 and 0.05 level.

The genotypes and treatment differed significantly (*P* ≤ 0.01) for the number of pods per plant and genotypes also behaved in distinct ways under different doses of NaCl as a relationship between genotypes and treatment was significant at *P* ≤ 0.01. Okra crop reduced the number of pods under saline condition. In mungbean, reduction was observed in different plant parameters under varying salinity levels^41, 42^. These results were also supported by Elshaikh et al.^31^ who reported that increasing soil salinity caused a significant decrease in plant growth and yield characteristics.

Salinity significantly (*P* ≤ 0.01) reduced the average fresh pod length and fresh pod diameter of all evaluated okra genotypes. The interaction between genotypes and treatment was highly significant which acknowledged that all okra genotypes behaved differently by increasing the concentration of NaCl. These results were in accordance with Fattah et al.^43^ who demonstrated that the pod length of okra was inversely related to salinity. The results are also in agreement with Bhadana et al.^44^ that by increasing the salinity level, the fresh pod diameter of okra decreased accordingly. The genotypes differed significantly (*P* ≤ 0.01) for stem girth of okra germplasm, and the interaction between genotypes and treatment of NaCl concentration was shown to be significant at *P* ≤ 0.01 (Table 2). These results were in accordance with Abbas et al. [42] who reported that the stem diameter of the okra plant showed a negative response to higher amount of salinity in irrigation water. Leaf area is a very important character that differed significantly (*P* ≤ 0.01) among okra genotypes under the varying concentration of NaCl. By changing the treatment level, the response of genotypes differed significantly at *P* ≤ 0.01 (Table 2). Our results are in accordance with Munns^45^ who found that salinity-induced decline in cell division and cell elongation as well as an injury due to specific ion toxicity were the main reasons for reduced leaf area under saline conditions. Latrach et al.^46^ also added that due to reduced leaf area under saline conditions, the rate of photosynthesis decreased, causing a severe decline in overall growth and yield. It was further reported that higher accumulation of Na + and Cl − under saline conditions could be the main reason for reduced leaf area. The identification of salt-tolerant and salt-susceptible genotypes within Okra germplasm signifies not only a scientific milestone but also a transformative advancement for sustainable agriculture. These insights empower precision breeding, underscore the importance of genetic diversity, and bear the potential to address the challenges of salt-affected soils while promoting broader agricultural resilience, economic prosperity, and food security. This scientific pursuit represents a monumental stride toward the realization of sustainable and resilient agricultural systems.

### Physiological characteristics

Relative water contents (RWC) of okra genotypes were significantly (*P* ≤ 0.01) influenced by NaCl concentration. Salinity stress reduced the RWC due to salinity-induced osmotic shock which inhibited the movement of water from the soil to plant. The reduction in RWC of okra genotypes under salinity stress holds significant physiological implications for the plants, impacting their overall health and performance. Limited movement of water from soil to plant reduces the cell turgidity. This, in turn, prompts stomatal closure to reduce water loss through transpiration, affecting photosynthesis by limiting carbon dioxide entry. As a result, there is a reduced production of carbohydrates and energy for plant growth. Furthermore, nutrient uptake is disrupted, and the plants become more vulnerable to diseases and pests. The decreased water use efficiency due to reduced RWC results in yield loss, with smaller and fewer fruits being produced. Prolonged salinity stress can lead to stunted growth, and the overall health and vitality of the okra plants are compromised^5, 46^. So, the water potential of the plant was highly influenced by salt stress conditions^48^. Many important crops like alfalfa, pea, balm, tomato, and cotton showed reduced RWC under saline conditions which ultimately caused a huge economic loss in the form of yield loss^49–51^. Salinity stress had a significant (*P* ≤ 0.01) effect on the EL of all genotypes (Table 2). A remarkable difference was observed in genotype × treatment interaction which remained significant at *P* ≤ 0.01. The difference in EL of okra genotypes in response to salinity showed their genetic potential to tolerate salinity, higher EL depicted lower salt tolerance^52^. An increase in EL by increasing salinity has also been reported by Salwa et al.,^53^ in peanuts; Latrach et al.,^46^ in alfalfa; Baninasab and Baghbanha^54^, in cucumber. This complex interplay of physiological changes underscores the need for effective strategies to mitigate the effects of salinity stress in affected areas, such as improved irrigation practices, the use of salt-tolerant crop varieties, and soil management techniques.

### Ionic concentration and ratio

By increasing external NaCl concentration in the growth medium, the leaf Na^+^ concentration increased differently in different genotypes, and the difference among the genotypes was found significant (*P* ≤ 0.01) (Table 2). The relationship between genotypes and treatment was highly significant which showed that genotypes performed differently concerning Na^+^ concentration under saline conditions. Abbas et al.^27^reported that under salt-stressed conditions, tolerant cultivars of okra germplasm restrained Na^+^ in their root zone and carried a low concentration of Na^+^ in their leaves. In okra plants, the salt tolerance mechanism might be associated with the efficient exclusion of Na^+^ as mentioned by Flowers & Yeo^55^ in rice.

Salinity stress conditions significantly (*P* ≤ 0.01) and differently affected the K^+^ concentration of all okra genotypes. By increasing NaCl concentration externally, K^+^ concentration significantly decreased in okra leaves and a significant difference was observed among all genotypes. Also, there was a significant relationship between genotypes and NaCl treatment (*P* ≤ 0.01). The reduction in leaf K + with increasing salinity was due to the antagonistic effect of Na^+^ on K^+^. Since under saline conditions increased concentration of Na^+^ inhibited the uptake of K^+^ as uptake and transport carriers for Na^+^ and K^+^ are common. However, the degree of reduction varied with genotypes which was due to the differential affinity of genotypes for Na^+^ and K^+^. The variations in leaf Na^+^ concentration and the K + :Na^+^ ratio among different okra genotypes under salinity stress highlight the diversity of salt tolerance mechanisms. Genotypes that can effectively exclude Na^+^ from their leaves and maintain a higher K^+^:Na + ratio are better equipped to thrive in saline conditions. These findings are essential for identifying and breeding salt-tolerant okra cultivars. The high affinity of genotypes for K^+^ compared to Na + could be an important salt tolerance mechanism^7, 56^. Shahid et al.^57^ revealed that by increasing NaCl concentration, a clear-cut reduction was noticed in K^+^ concentration in plant tissues, and higher K^+^ concentration was closely related to salt tolerance.

Leaf K + : Na^+^ ratio was affected significantly (*P* ≤ 0.01) by adding NaCl concentration externally (Table 2). Genotype into treatment interaction differed significantly at *P* ≤ 0.01 among all growing okra genotypes. Increased level of NaCl salinity in the growth medium increased the concntration of Na^+^ while simultaneously decreasing K^+^, resulting in a decline in K^+^: Na^+^ ratio. The differences among okra genotypes for K^+^: Na^+^ ratio were due to their preferential uptake of K^+^ over Na^+^ under saline conditions^27^. Na^+^ exclusion and K^+^ uptake are vital components of okra plants' salt tolerance mechanisms. Genotypes proficient in excluding Na + and maintaining a favorable K^+^:Na + ratio thrive in saline conditions. These mechanisms preserve cellular integrity, support normal physiological processes, and enhance okra growth and productivity in salt-affected regions. Leveraging these mechanisms can help develop salt-tolerant okra varieties and promote sustainable agriculture in saline environments.

### Principal component analysis

Principal component analysis (PCA) is an important technique used to observe variation among multiple accessions and traits. PCA model was also used, to sum up various quality parameters into one major quality index to promote the quality of okra. PCA can also be used for the assessment of the independent effect of the specific parameter over the total variance observed such as the coefficient of proper vector showed the contributed index of every original variable with the association of each principal component^58, 59^. PCA results provide valuable insights into the selection and breeding of okra genotypes with desired traits. By focusing on the traits that contribute the most to variance and considering trait associations, breeders can develop more effective strategies for enhancing okra quality and overall performance. According to Chen et al. [60], the first, three principal components are highly significant and widely used as variation patterns among various accessions. PCA allows for the development of a quality index, which condenses multiple traits into a single metric. This simplifies the evaluation of okra quality and facilitates the comparison and selection of genotypes based on a comprehensive quality measure.

### Principal component analysis for normal condition (control)

In this study, PCA was performed to get information about how to select okra genotypes with desired traits in normal conditions. Means data were used to perform principal components analysis for the traits which showed significant results. Under normal conditions, PCA was conducted on the total variability of morpho-physiological traits in okra genotypes, resulting in 16 principal components (as presented in Table 3). which”’Among these components, the first 6 PCs contributed the most to the total variation, each with an eigenvalue greater than one, indicating a broad genetic diversity within the studied okra germplasm. The first six PCs contributed 67% cumulated variation in okra accessions while 33% variation was contributed by the remaining 10 PCs. The principal component analysis is an important tool used to distinguish genotypic responses for varying levels of salinity^36, 61^. PCA findings under normal conditions provide a foundation for understanding trait prioritization and the performance of okra genotypes in non-saline environments. Comparing these results with findings from saline conditions allows breeders to assess how genotypes respond to salt stress and identify those with consistent quality across varying environments. It also reveals trade-offs and correlations between traits and assists in selecting adaptable genotypes for breeding programs. Ultimately, this approach helps in developing okra varieties that thrive under different conditions, addressing the challenges of salinity and enhancing overall quality and productivity.

**Table 3:** Eigen value and percent total variance for principle components for under normal condition.

Principal components	Eigen value	Variability percentage	Cumulative percentage
PC1	3.6	21.4	21.4
PC2	2.5	14.5	35.9
PC3	1.6	9.5	45.4
PC4	1.4	8.4	53.7
PC5	1.3	7.4	61.1
PC6	1.0	6.1	67.2
PC7	0.9	5.5	72.6
PC8	0.8	4.9	77.6
PC9	0.8	4.6	82.2
PC10	0.7	4.3	86.5
PC11	0.5	3.2	89.7
PC12	0.5	3.1	92.8
PC13	0.4	2.6	95.4
PC14	0.4	2.3	97.8
PC15	0.2	1.5	99.2
PC16	0.1	0.7	100

### Contribution of plant traits to genetic variability under normal conditions

**Figure 1:**
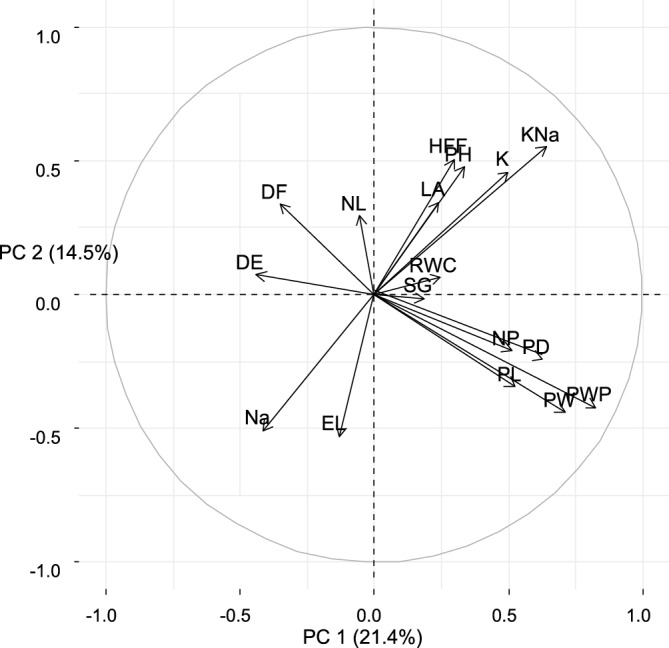
Contribution of plant traits towards genetic variation in studied okra germplasm.

The contribution of studied traits for genetic variability is presented in Fig. 1. Biplot analysis for morpho-physiological parameters of both PC1 and PC2 revealed that stem girth, RWC, leaf area, height at the first flower, plant height, K + , and K + /Na + were the most important traits and contributed significant genetic variation in studied okra germplasm. While the share of DTE, DTF, and the number of leaves at a first flower, Na + and EL were minimum in genetic variation. K + and K + : Na + ratio showed positive and significant association with height at first flower, number of pods, and pod weight but negative and significant association with EL and Na + .

### Bi-plot analysis

**Figure 2:**
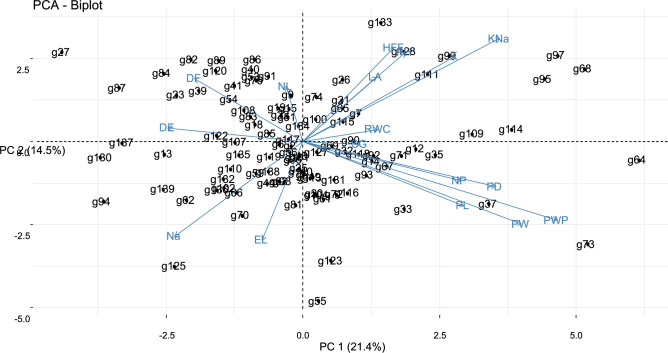
Bi-plot analysis representing 17 plant traits in okra germplasm for normal condition.

Bi-plot analysis for the contribution of okra germplasm based on studied plant traits is presented in Fig. 2. It revealed that okra genotypes Silky-360, Clemson, and Acc-15379 showed maximum variation for height at first flower and overall plant height, while Acc-15376, Acc-34398, and Ok-135 contributed minimum share to create variation due to their location near the base point. Genotypes Sanura and Peted revealed broad genetic diversity for K + and K + : Na + because these accessions remained at a distant location from the origin of the bi-plot. However, accession Ok-1300 and Acc-15366 contributed okra accessions were located at a distant position from starting point of the bi-plot whereas, genotypes 93, 131, 116, and 72 contributed the smallest share for the development of genetic variation due to their position at the origin of bi-plot. Genotypes 91, 99, 15, 19, 82, 39, 84, 23, 137 showed significant associations for node length, DTF and DTE. Similarly, accessions 62, 125, 70, and 81 revealed a positive relationship with Na + and EL. The integrity of the PCA bi-plot makes it more predictable than other statistical tools for the evaluation of all genotypes under saline conditions [27] the slightest share for genetic variation because these genotypes were placed at starting point of the bi-plot. Due to the farthest placement maximum genetic variation was calculated for stem girth and RWC regarding Acc-15365, Acc-20246, Acc-36962, Parbhani, and Acc-19223. Meanwhile, Acc-34402 and Acc-1582 had little share to produce genetic variation, however, these accessions were found near the center of origin of the bi-plot. Accession 20,376 exhibited the greatest variation for the number of pods and pod diameter while accession 34,400 revealed the lowest genetic variation regarding these parameters due to the presence near the base point. Similarly, Acc-34406, Acc-20376 and Acc-20297 displayed maximum genetic variation for pod length, pod weight plant-1, fresh pod weight because these okra accessions were located at a distant position from starting point of the bi-plot whereas, genotypes 93, 131, 116, and 72 contributed the smallest share for the development of genetic variation due to their position at the origin of bi-plot. Genotypes 91, 99, 15, 19, 82, 39, 84, 23, 137 showed significant associations for node length, DTF and DTE. Similarly, accessions 62, 125, 70, and 81 revealed a positive relationship with Na + and EL. The integrity of the PCA bi-plot makes it more predictable than other statistical tools for the evaluation of all genotypes under saline conditions [27]

### Cluster analysis of okra germplasm under normal conditions (control)

Cluster analysis graded okra genotypes into five clusters (cluster-1 to cluster-5) based on genetic diversity present in germplasm under studied morpho-physiological parameters (Table 4). Cluster-1 represented 18 genotypes based on high yield while cluster-2 displayed 24 genotypes that showed minimum pod yield. The remaining clusters such as cluster-3, cluster-4, and cluster-5 comprised 23, 18, and 17 okra accessions, respectively showing a moderate contribution to the genetic diversity of okra germplasm. All the above said clusters showed that okra genotypes performed similar or nearly the same response within the cluster but exhibited different behavior across the cluster.

**Table 4:** Grouping of okra genotypes by means of cluster analysis.

Cluster	No of Genotypes	Genotype
1	18	G119, G69, G70, G138, G35, G13, G33, G144, G140, G101, G110, G20, G108, G63, G137, G139, G94, G125
2	24	G97, G114, G109, G12, G98, G90, G93, G59, G41, G84, G116, G136, G122, G49, G135, G6, G83, G81, G123, G92, G32, G19, G96, G130
3	23	G64, G99, G117, G51, G74, G65, G46, G67, G55, G56, G113, G123, G61, G82, G15, G91, G18, G102, G100, G89, G54, G53, G87
4	18	G111, G118, G107, G104, G37, G7, G21, G71, G9, G62, G66, G25, G14, G76, G86, G131, G132, G127
5	17	G68, G95, G128, G133, G72, G120, G73, G22, G85, G39, G5, G2, G40, G26, G134, G127, G115

#### Cluster 1

Cluster-1 comprised okra genotypes 64, 73, 37, 35, 55, 109, 97, 118, 68, and 95 which showed maximum genetic diversity for okra pod yield. Although, all these accessions exhibited a narrow genetic base within the cluster while considered as high yielder breeding lines when compared with other clusters for plant traits such as height at first flower, plant height, K + , K + : Na + ratio, leaf area, RWC, and stem girth.

#### Cluster 2

Cluster 2 exposed narrow range of genetic variation for okra accessions 23, 134, 40, 130, 02, 84, 82, 120, 87 as well as 27. All these okra genotypes were declared as overall low yielders, while performing well by producing the maximum number of leaves at first flower, and took minimum DTF as well as minimum DTE.

#### Cluster 3

Okra genotypes under cluster 3 showed higher values for quality parameters. It included genotypes 70, 94, 137, 139, and 125 which gained maximum values for Na + concentration. Genotypes 119, 69, 138, 35, 13, 33, 144, 140, 101, 110, 20, 108, and 63 also performed better for other morpho-physiological parameters in this cluster.

#### Cluster 4

Cluster 4 consisted of okra accessions 111, 118, 107, 104, 37, 7, 21 71, 9, 62, 66, 25, 14, 76, 86, 131, 132 and 127 showed moderate behavior to create genetic diversity under normal condition.

#### Cluster 5

Cluster 5 comprised of 17 okra genotypes, presented maximum value for morho-physiological parameters in studied okra germplasm Table 5. In this cluster, okra genotypes 68, 95, 128, and 133 performed better for K + : Na + ratio, height at first flower, and overall plant height, while genotypes 72, 120, and 73 showed maximum variability for pod weight plant-1 and pod weight. Genotypes 22, 85, 39, 5, 2, 40, 26, 134, 127 and 115 showed significant role for other quality parameters.

**Table 5:** Mean values of morphological and physiological traits of okra germplasm under five clusters.

Cluster no	1	2	3	4	5
DE	8.05	8.82	9.04	9.27	8.15
DF	53.92	56.72	58.98	57.03	54.31
NL	3.78	3.57	3.76	3.59	3.98
HFF	10.57	10.49	11.54	11.27	12.84
NP	6.91	6.93	6.59	6.45	6.39
PWP	32.99	35.53	32.63	33.43	33.44
PW	4.76	5.14	4.91	5.12	5.17
PL	6.42	6.52	6.17	6.37	6.71
PD	12.01	12.59	12.47	12.67	12.67
SG	10.75	10.54	10.73	11.22	11.61
LA	2344.70	4206.72	5217.57	3204.62	6897.21
EL	84.39	81.56	81.63	81.91	8053.58
RWC	70.15	72.94	77.66	71.91	77.69
PH	39.32	43.26	43.79	49.29	47.77
Na	8.34	7.87	8.07	7.05	6.62
K	30.45	39.80	40.14	37.13	36.77
Na/K	4.22	6.17	5.92	6.03	6.45

### Optimal number of clusters

**Figure 3:**
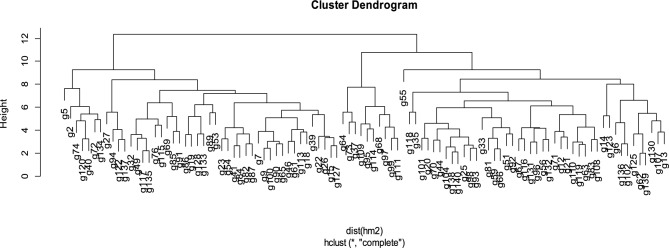
Grouping of okra accessions in different groups under normal condition.

Total variations were shown in 10 clusters. But maximum variation occurred up to cluster number 5. Graph (Fig. 3) showed that maximum variation was recorded in cluster number 1 and minimum genetic variation was noticed in cluster number 10. It also revealed from the curve that whenever we moved from cluster 2 to 3 and onward downward curve exhibited a narrow genetic base.

### Principal component analysis for stress condition

In this research principal component analysis (PCA) was conducted to get facts about how to select okra accessions for preferred traits in a salt-stressed regime. PCA was done by using mean values for those traits which showed significant genotypes and treatment interactions. Under the stressed condition, PCA distributed the total variability of okra germplasm for morpho-physiological parameters into 16 components presented in Table 6. Among these components, the first five PCs showed supreme variation having eigenvalues of more than one which revealed broad genetic variability for okra germplasm. The first five principal components added 67% cumulated variation in okra genotypes however remaining PCs contributed 33% genetic variability in okra germplasm.

**Table 6:** Principal component of 100 okra genotypes under salinity stress.

Principal components	Eigen value	Variability percentage	Cumulative percentage
PC1	6.7	39.2	39.2
PC2	1.4	8.2	47.4
PC3	1.2	7.2	54.6
PC4	1.2	7.1	61.7
PC5	1.0	6.0	67.7
PC6	0.9	5.1	72.7
PC7	0.8	4.7	77.4
PC8	0.7	4.0	81.4
PC9	0.6	3.8	85.2
PC10	0.6	3.7	88.9
PC11	0.5	2.8	91.6
PC12	0.4	2.5	94.2
PC13	0.4	2.1	96.3
PC14	0.3	1.7	98.0
PC15	0.3	1.7	99.6
PC16	0.1	0.3	100

### Contribution of plant traits to genetic variability under stressed condition

**Figure 4:**
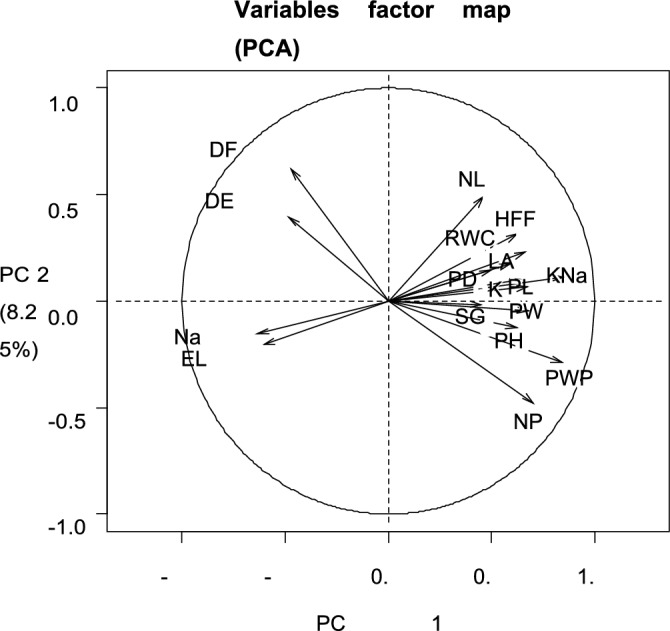
Contribution of plant traits towards genetic variation in studied okra germplasm under stress.

Bi-plot analysis for morpho-physiological parameters of both PC1 and PC2 presented in Fig. 4 showed that the number of leaves, height at first flower, RWC, leaf area, pod diameter, K + : Na + ratio, K + concentration, pod length, pod weight, stem girth, plant height, pod weight plant-1 and number of pods were the most important parameters which contributed significant genetic variations in studied okra accessions. However, DTF, DTE, Na + , and EL contributed a minimum share of genetic variations. Na + and EL showed a positive association with DTF and DTE but a negative significant association with all other yield-contributing and salinity-tolerant traits under study. The differences in salt tolerance among different okra genotypes can be attributed to various traits and physiological processes that interact to determine their responses to salinity. Understanding these mechanisms is essential for developing salt-tolerant okra cultivars. The potential mechanisms and factors that may contribute to the observed differences in salt tolerance among okra genotypes are ion transport and regulation, osmotic adjustment, antioxidant enzymes, stomatal regulation and metabolic pathway^34, 52^. The variation in salt tolerance among okra genotypes is the result of complex interactions between genetic, physiological, and biochemical factors. Identification and selection of genotypes with superior traits in the context of these mechanisms can aid in the development of salt-tolerant okra cul-tivars, contributing to food security in regions affected by salinity^4^.

### Bi-plot analysis

**Figure 5:**
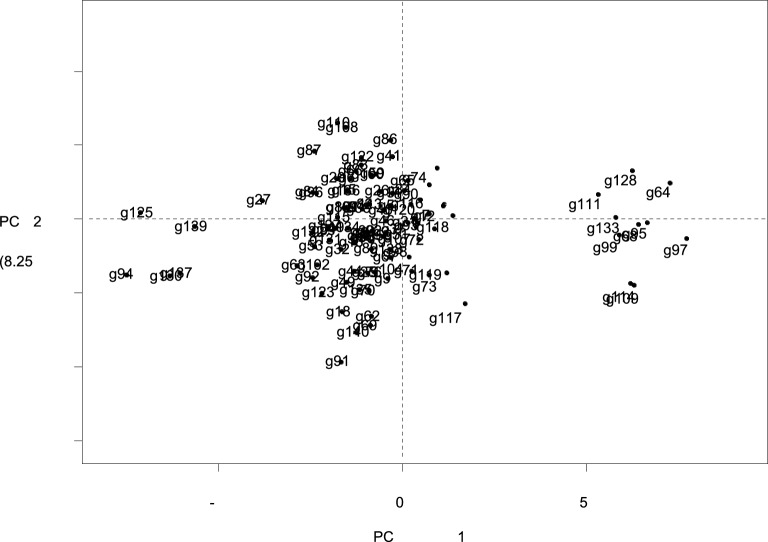
Bi-plot analysis representing plant traits in okra germplasm for stressed condition.

Among all other parameters, RWC, height at first flower, and the number of leaf at first flower revealed the highest genetic variation for screening of okra germplasm under salt stress conditions for genotypes 111, 128, and 64 (Fig. 5). Screening of the okra breeding line was also based on the leaf area of plants because under saline conditions, leaf area was decreased as a result of the decline in photosynthetic activity for accession 133 and 95, these genotypes also negatively associated with Na + concentration and EL for genotypes 94, 137 and 30. Pod yield is the ultimate purpose which was governed by pod diameter, accession No. 133 had a maximum genetic variation for pod diameter. Similarly, maximum pod length, K + , K + : Na + was calculated for genotypes 68, 95, and 99 collectively. Genotypes 125, 139, and 27 took maximum DTE and DTF so they showed a negative relationship for these traits. Cell membrane injury is also an important parameter for the evaluation of salt tolerance. It is a widely known fact that salinity enhances osmotic stress that deteriorates the cellular activities of crop plants (Munns 2001). Concentrations of K + negatively correlated with EL^62^as it inhibited the uptake of Na + , and reduced EL. PC bi-plot also illustrated the antiparallel vector position of EL and K + : Na + . EL was used as a valuable tool to evaluate salt tolerance in breeding programs^63^because it was positively correlated with the accumulation of Na + in plant cells.

### Cluster analysis

Cluster analysis is an exploratory analysis that tries to identify clusters within the data. Cluster analysis is also known as segmentation analysis. Under stress conditions cluster dendrogram divided genotypes into five major groups (cluster 1- cluster 5). Cluster 1 contains 24 genotypes, cluster 2 consists of 26 breeding lines cluster 3 to cluster 5 comprised of 6, 34 and 10 okra accessions, respectively represented in Table 7.

**Table 7:** Grouping of okra genotypes by means of cluster analysis.

Cluster	No. of genotypes	Genotype
1	24	G90, G93, G65, G41, G84, G116, G122, G26, G15, G14, G100, G89, G6, G83, G86, G110, G32, G54, G20, G108, G19, G96, G87, G115,
2	26	G12, G117, G72, G118, G73, G74, G119, G71, G69, G9, G67, G55, G62, G5, G70, G135, G25, G138, G44, G91, G140, G18, G76, G134, G123, G53
3	6	G27, G137, G139, G130, G94, G125
4	34	G51, G120, G98, G107, G104, G37, G7, G21, G22, G46, G85, G59, G39, G56, G113, G66, G2, G40, G23, G136, G61, G35, G49, G13, G82, G33, G102, G101, G81, G131, G127, G92, G132, G63
5	10	G97, G64, G68, G95, G99, G114, G111, G128, G109, G133

#### Cluster 1

**Figure 6:**
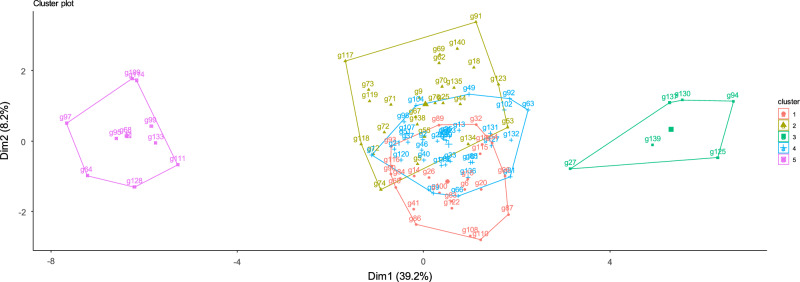
Cluster analysis showing five clusters arrangement under stress.

**Figure 7:**
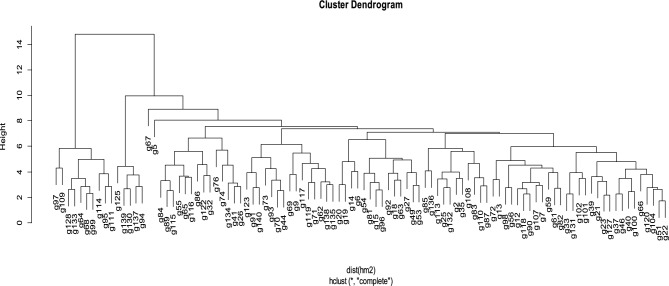
Dendrogram showing grouping of okra accessions under stress condition.

Cluster 1 showed that genotypes within the cluster had minimum genetic variability for genotypes 64, 68, 95, 97, 99, 109, 111, 114, 128, and 133 (Figs. 6 and 7). All these mentioned okra genotypes indicated better responses for salt-tolerant traits under stress conditions and were selected as tolerant genotypes (Table 8).

**Table 8:** Mean values of morphological traits of okra germplasm under five clusters.

Cluster No	1	2	3	4	5
DE	0.86358	− 0.34839	0.74704	− 0.11199	− 1.23424
DF	0.81759	− 0.14159	1.05435	− 0.26097	− 1.33936
NL	0.08624	− 0.86875	− 0.86356	0.51070	1.24748
HFF	0.16615	− 0.09713	− 1.74353	0.12773	1.26315
NP	0.53655	0.42365	− 0.96215	− 0.33279	1.89502
PWP	0.40306	− 1.50030	− 0.30548	2.04685	0.48166
PW	0.28381	0.37532	− 1.86593	− 0.13354	1.27893
PL	0.01796	− 0.27076	− 1.51141	− 0.02310	1.64627
PD	0.11073	− 0.00093	− 1.51941	− 0.07501	0.90383
SG	0.08461	0.29151	− 1.45939	− 0.14454	0.81221
LA	0.11147	− 0.19098	− 0.94843	− 0.24477	1.63033
EL	0.25754	0.34196	0.65368	0.35048	− 1.85486
RWC	0.28146	− 1.77987	0.16228	1.40738	0.06642
PH	0.24222	0.02532	− 1.22741	− 0.08769	1.55010
Na	0.03828	− 0.24371	1.36365	0.36883	− 1.34667
K	0.55477	− 0.31591	− 1.54269	− 0.35918	1.63677
Na/K	0.21296	− 0.21436	− 1.20233	− 0.49682	2.45684

#### Cluster 2

Genotypes present in cluster 2 exhibited minimum genetic variation for okra genotypes 27, 94, 125, 139, 130, and 137. All these genotypes showed significant genetic variability with other genotypes in okra germplasm for salt tolerance-related traits having poor performance and acting as salt susceptible genotypes. Broad genetic variability was present between cluster A and cluster B.

#### Cluster 3

Cluster 3 comprised of 24 okra genotypes. Genotypes 6, 14, 15, 19, 20, 26, 32, 41, 54, 65, 83, 84 and 86 might be stimulated due to increasing saline condition (Table 8). Accessions 87, 89, 90, 93, 96, 108, 100, 110 exhibited positive relationships with DTE and DTF. Genotypes 115, 116 and 122 performed better under saline regimes.

#### Cluster 4

Cluster 4 consisted of 26 okra genotypes. In order to enhance genetic variability sufficient variations were also observed in genotypes 5, 9, 12, 18, 25, 44, 53, 55, 62, 67, 69, 70, 71, 72, 73, 74, 76, 91, 117, 118, 119, 123, 134, 135, 138 and 140.

#### Cluster 5

Cluster 5 contained of 34 okra genotypes included genotype 51, 120, 98, 107, 104, 37, 7, 21, 22, 46, 85, 59, 39, 56, 113, 66, 2, 40, 23, 136, 61, 35, 49, 13, 82, 33, 102, 101, 81, 131, 127, 92, 132 and 163.

#### Optimal number of clusters

**Figure 8:**
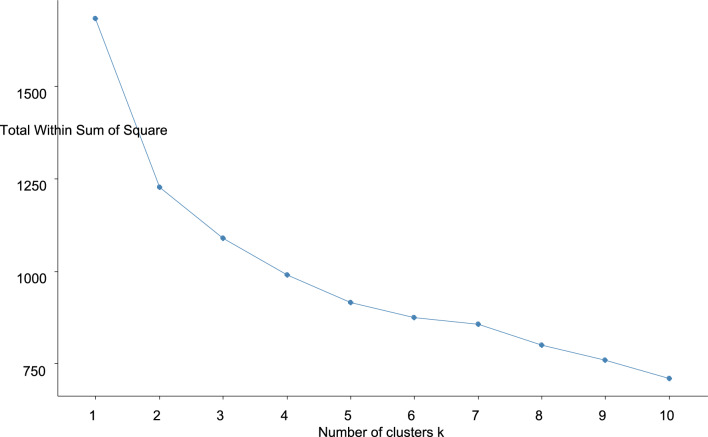
Optimal number of cluster for genetic diversity in okra germplasm under stress.

Genotypes showing the same response are graded into the same cluster. Optimal number of clusters is inversely related to the genetic variation found in okra germplasm. Clusters 1 and 2 exploited the maximum genetic diversity, and cluster 3, 4 and 5 showed narrow genetic variations (Fig. 8).

## Correlation

### Under normal condition

Correlation analysis is a very important statistical approach used to find out the association between two or more two variables. When the shift of one variable occurs along by the movement of another variable, that type of variable is said to be correlated with each other.

Table 9 reveals the correlation among 17 quantitative parameters in the studied okra genotypes. If a single character increases and is used for selection purposes, the positively correlated trait will also be selected and vice versa. DTE had a positive relationship with DTF, EL, and Na + concentration at the genotypic level under normal conditions. A significant positive association was observed for number of leaf at first flower, height at the first flower, stem girth, and leaf area. The number of leaf at first flower was linked positively with height at first flower, stem girth, leaf area, EL, RWC, plant height, and K + : Na + ratio. Height at first flower displayed a significant positive association with pod weight, pod weight per plant, pod length, pod diameter, stem girth, leaf area, RWC, and plant height, K + and K + : Na + . NP showed positive associations with all growing parameters except stem girth and leaf area. Pod weight showed a negative correlation with the concentration of Na + while other parameters exhibited a positive association. Pod weight per plant revealed a positive linkage with all parameters except Na + and plant height which displayed a negative relationship. Pod length, diameter, and stem girth indicated a positive association with all developing parameters except Na + .

**Table 9:** Correlations among 17 traits of okra germplasm under normal conditions.

Variables	DTE	DTF	NL	HFF	NP	PW	PWP	PL	PD	SG	LA	EL	RWC	PH	Na +	K +
DTE	1															
DTF	0.293	1														
NL	− 0.070	0.245	1													
HFF	− 0.069	0.131	0.265	1												
NP	− 0.334	− 0.324	− 0.029	− 0.010	1											
PW	− 0.333	− 0.320	− 0.095	0.073	0.709	1										
PWP	− 0.171	− 0.179	− 0.146	0.078	0.130	0.779	1									
PL	− 0.072	− 0.265	− 0.089	0.090	0.075	0.477	0.615	1								
PD	− 0.209	− 0.093	− 0.017	0.074	0.114	0.532	0.659	0.307	1							
SG	− 0.100	0.050	0.012	0.059	− 0.022	0.141	0.181	− 0.026	0.218	1						
LA	− 0.018	0.031	0.075	0.271	− 0.089	0.001	0.067	0.118	0.103	0.086	1					
EL	0.048	− 0.006	0.010	− 0.224	0.030	0.081	0.115	0.088	0.037	0.083	− 0.143	1				
RWC	− 0.099	− 0.050	0.109	0.204	0.171	0.186	0.116	0.035	0.126	− 0.124	0.325	− 0.049	1			
PH	− 0.144	− 0.073	0.016	0.337	0.130	0.086	− 0.014	0.044	0.009	0.093	0.122	− 0.308	0.071	1		
Na +	0.104	− 0.072	− 0.072	− 0.300	0.000	− 0.081	− 0.113	− 0.026	− 0.201	− 0.136	− 0.124	0.055	0.024	− 0.283	1	
K +	− 0.059	− 0.089	− 0.031	0.136	0.226	0.206	0.094	0.128	0.102	− 0.037	0.175	− 0.354	0.008	0.213	− 0.265	1
K + : Na +	− 0.203	− 0.059	0.031	0.239	0.218	0.270	0.179	0.086	0.239	0.071	0.210	− 0.255	0.046	0.329	− 0.667	0.727

Values in bold are different from 0 with a significance level alpha = 0.05. (DTE: Days to emergence; DTF: Days to flowering; NL: No. of leaves at first flower; HFF: Plant height at first flower; NP: No. of pods per plant; PW: Fresh pod weight; PWP: Fresh pod weight per plant: PL: Fresh pod length; PD: Fresh pod diameter; SG: Stem girth; LA: Leaf area; EL: Electrolyte leakage; RWC: Relative water contents; PH: Plant height; Na^+^: Sodium; K^+^: Potassium).

#### Under stress condition

DTE revealed a negative association with all morpho-physiological traits except DTF, EL, and Na + concentration because excessive Na + in the growth medium delayed the germination and emergence of plants^8^. The number of leaf, height at first flower, No. of pods, pod weight, pod weight per plant, pod length, pod diameter, and stem girth indicated a positive correlation with all emerging parameters except EL and Na + concentration (Table 10). Under salt-stressed conditions, a positive relationship was noticed between plant height and pod yield which indicated that selection for vigorous plants would earn greater yield potential. The positive association between pod yield and plant height might be some good criteria for the development of high-yielding okra germplasm. In past, many researchers reported a similar category of a positive association between pod yield and plant height^64, 65^. A highly significant correlation was also observed for plant height with pod yield plant-1 by Shakoor et al.^66^. Hence, selection for such traits compiles desirable genes which ultimately improved the yield potential in many crop plants.

**Table 10:** Correlations among 17 traits of okra germplasm under NaCl stress conditions.

Variables	DTE	DTF	NL	HFF	NP	PW	PWP	PL	PD	SG	LA	EL	RWC	PH	Na +	K +
DE	1															
DF	0.377	1														
NL	− 0.139	− 0.106	1													
HFF	− 0.281	− 0.140	0.535	1												
NP	− 0.379	− 0.534	0.143	0.311	1											
PW	− 0.380	− 0.459	0.287	0.432	0.816	1										
PWP	− 0.249	− 0.242	0.286	0.406	0.367	0.827	1									
PL	− 0.232	− 0.268	0.317	0.313	0.349	0.561	0.569	1								
PD	− 0.125	− 0.219	0.176	0.326	0.227	0.328	0.330	0.411	1							
SG	− 0.278	− 0.092	0.066	0.312	0.281	0.318	0.259	0.141	0.188	1						
LA	− 0.174	− 0.179	0.208	0.292	0.287	0.439	0.404	0.296	0.285	0.276	1					
EL	0.240	0.144	− 0.313	− 0.292	− 0.375	− 0.387	− 0.232	− 0.318	− 0.089	− 0.265	− 0.395	1				
RWC	− 0.185	− 0.214	0.408	0.388	0.434	0.475	0.353	0.454	0.349	0.127	0.437	− 0.381	1			
PH	− 0.329	− 0.323	0.192	0.424	0.438	0.452	0.311	0.404	0.233	0.270	0.326	− 0.322	0.424	1		
Na	0.329	0.114	− 0.219	− 0.404	− 0.332	− 0.444	− 0.394	− 0.355	− 0.346	− 0.452	− 0.345	0.307	− 0.441	− 0.349	1	
K	− 0.183	− 0.305	0.246	0.319	0.397	0.427	0.303	0.424	0.318	0.190	0.351	− 0.570	0.333	0.264	− 0.166	1
K/Na	− 0.363	− 0.336	0.338	0.451	0.524	0.591	0.415	0.529	0.396	0.375	0.479	− 0.638	0.480	0.449	− 0.629	0.791

Values in bold are different from 0 with a significance level alpha = 0.05. (DTE: Days to emergence; DTF: Days to flowering; NL: No. of leaves at first flower; HFF: Plant height at first flower; NP: No. of pods per plant; PW: Fresh pod weight; PWP: Fresh pod weight per plant: PL: Fresh pod length; PD: Fresh pod diameter; SG: Stem girth; LA: Leaf area; EL: Electrolyte leakage; RWC: Relative water contents; PH: Plant height; Na^+^: Sodium; K^+^: Potassium).

## Materials and methods

### Collection of germplasm

Okra germplasm of 100 genotypes was randomly collected from various research institutes and stations in Pakistan including the Plant Genetic Resources Program (PGRP) National Agricultural Research Center (NARC), Islamabad, Vegetable Research Station, Ayub Agriculture Research Institute (AARI), Faisalabad and the University of Agriculture, Faisalabad. This study adheres to the institutional, national, and international guidelines and legislation.

### Screening of germplasm

Germplasm screening was conducted in the research area of the Department of Plant Breeding and Genetics, College of Agriculture, University of Sargodha, Sargodha, Pakistan (32 0.07°, Latitude N, 74.41°, E Longitude and Altitude 189 m). The pot experiment was conducted during spring 2019 in open environment and irrigated twice a week to fulfill the evapotranspiarational requirements of plants. The surface sterilized seeds with 0.2% hypochlorite of 100 okra genotypes were sown in duplicate in earthen pots filled with 10 kg silt soil lined with polythene sheet to avoid irrigation water and nutrient losses. The seeds of 100 okra genotypes were thoroughly rinsed with tap water after hypochlorite treatment to remove residues, and sown under two conditions:, control and 6.5 dS m^−1^ NaCl concentration conditions The salinity was induced using sodium chloride (NaCl), following the procedure developed by U.S. Salinity Laboratory Staff in 1954. According to the mthod, required amount of NaCl was mixed during pot filling stage to achieve the salinity level of 6.5 dS m^−1^. Five okra seeds were sown in each pot of both conditions (control and salinity stress) and three uniform plants were kept in each pot after thinning. The plants were grown to maturity ”sing’a completely randomized design (CRD) with three replications. Half-strength Hoagland nutrient solution was added to pots once in a week to fulfill the nutrients requirement of plants.

### Measurement of morphological characteristics

Data for various morphological parameters, including days to emergence (DTE), days to flowering (DTF), plant height at first flowering, number of pods per plant, pod length, pod diameter were recorded.”

### Measurement of Physiological characteristics

To determaine the RWC, healthy plant leaves, preferably the 3^rd^ topmost leaves, were selected during the 6th week after sowing when they reached the desired growth stage. First, the leaf’s fresh weight (FW) were immediately measured using a precision balance upon collection. This served as a baseline for further calculations. Subsequently, the leaves were subjected to determine their turgid weight (TW). The leaves were submerged in distilled water for a period of 4 h to ensure full hydration. After this period, the leaves were removed, and their surface was carefully blotted with tissue paper to eliminate excess water. The turged weight of leaf was then recorded. In next step oven dried weight (ODW) of leaves were determined. To prevent contamination and moisture loss, the leaves were sealed in an airtight plastic bag and labeled for identification. The leaves were placed in an oven set at 70 °C for 48 h to ensure complete drying. After this period, the leaves were removed from the oven and allowed to cool within the sealed bag. The weight was measured using the precision balance, and this recorded weight represents the ODW of the leaf. RWC was determined according to Yamasaki and Dillenburg^67^.

$${\text{RWC }}\left( \% \right) = [\left( {{\text{FW}} - {\text{ODW}}} \right)/\left( {{\text{TW}} - {\text{ODW}}} \right] \times {1}00$$

### Electrolyte leakage%

For electrolyte leakage (EL), the 4th leaf from the shoot tip was collected from each pot during 6th week after sowing. The collected leaves were cut into 1.0 cm diameter pieces and put in test tubes having 10 mL deionized water in two sets ensuring that leaves submerged completely. One set of test tubes was kept in water bath at 40 °C for 30 min and the EC of water having leaf sample was measured (C1). The electrode of EC meter was washed, wigh deionized water and blotted with tissue paper before moving to next tube. The second set of test tubes was autoclaved for 15 min, it was allowed to cool down at room temperature and EC was measured (C2) of each test tube containing separate genotypes leaf. Electrolyte leakage% was calculated according to Sairam^57^.

$${\text{Electrolyte leakage}}\, = \,\left[ {{\text{C1}}/{\text{C2}}} \right]\, \times \,{1}00$$

### Ion determination

Wet digestion was performed for the determination of sodium (Na^+^) and potassium (K^+^) concentrations. Briefly, 1 g of dried plant leaf material in powdered form was transferred into a 100 ml digestion conical flask. After adding a 10 mL di-acid mixture of nitric acid and perchloric acid in the ratio of 2:1, the conical flasks were placed on a hot plate at 250 °C for 30 min. The digested samples were diluted to 50 mL using distilled water. Na^+^ and K^+^ were determined in mmol L^−1^ using Flame Photometer (Jenway PFP7).

### Statistical analysis

Data collected for morpho-physiological parameters were subjected to analysis of variance (ANOVA). The traits that showed significant differences were further used to compare the means of various plant genotypes. The significance of means was compared by the Least Significance Differences test (LSD) using the software Statistics 8.1. The LSD test is used to compare the means of multiple groups, which identifies significant differences between the means of the groups. Mean data of the traits that showed significant genotype × environment interaction were subjected to principal component analysis (PCA) using R Studio, Inc. following the method described by Husson et al.^68^."

## Conclusions

The assessment of the genetic potential of Okra germplasm for salinity tolerance revealed distinct outcomes within the study. Notably, a wide range of genetic diversity was observed among the okra genotypes in their responses to salt stress conditions. This genetic diversity was evident in the performance of these genotypes concerning traits related to salt tolerance. In essence, the genetic diversity indicated that different genotypes exhibited varying degrees of resilience or susceptibility when confronted the salinity-induced stress. Among the observed genotypes, a subset, specifically genotypes 64, 68, 95, 97, 99, 109, 111, 114, 128, and 133, demonstrated remarkable and improved responses to salt-tolerant traits under the challenging conditions of salt stress. These genotypes exhibited the ability to thrive or maintain their vital functions in the presence of elevated salinity, indicating their suitability for salt-affected environments. As such, they were selected as the group of salt-tolerant genotypes due to their superior performance. Conversely, another set of genotypes, namely genotypes 27, 94, 125, 139, 130, and 137, displayed genetic diversity compared to the other genotypes within the okra germplasm. These genotypes were characterized by their poor performance in salt tolerance-related traits. They struggled to cope with the stress imposed by salinity and exhibited lower adaptability to such adverse conditions. Consequently, they were identified as salt-susceptible genotypes, signifying their vulnerability to salinity-induced challenges. Overall, this study underscores the significant genetic variability within the okra germplasm in response to salt stress, and it highlights the existence of distinct genotypes with varying degree of salt tolerance. This information is valuable for future breeding and selection efforts aimed at developing salt-tolerant okra cultivars, ultimately contributing to the sustainability and productivity of okra cultivation in salt-affected regions.

## Data Availability

The datasets generated and/or analyzed during the current study are not publicly available but will be available from the corresponding author on reasonable request.
